# Flavonoid Fraction of Bergamot Juice Reduces LPS-Induced Inflammatory Response through SIRT1-Mediated NF-κB Inhibition in THP-1 Monocytes

**DOI:** 10.1371/journal.pone.0107431

**Published:** 2014-09-26

**Authors:** Roberto Risitano, Monica Currò, Santa Cirmi, Nadia Ferlazzo, Pietro Campiglia, Daniela Caccamo, Riccardo Ientile, Michele Navarra

**Affiliations:** 1 Department of Drug Sciences and Products for Health, University of Messina, Messina, Italy; 2 Department of Biomedical Sciences and Morphofunctional Imaging, University of Messina, Messina, Italy; 3 Department of Pharmaceutical and Biomedical Sciences, University of Salerno, Fisciano, Salerno, Italy; University of Catania, Italy

## Abstract

Plant polyphenols exert anti-inflammatory activity through both anti-oxidant effects and modulation of pivotal pro-inflammatory genes. Recently, *Citrus bergamia* has been studied as a natural source of bioactive molecules with antioxidant activity, but few studies have focused on molecular mechanisms underlying their potential beneficial effects. Several findings have suggested that polyphenols could influence cellular function by acting as activators of SIRT1, a nuclear histone deacetylase, involved in the inhibition of NF-κB signaling. On the basis of these observations we studied the anti-inflammatory effects produced by the flavonoid fraction of the bergamot juice (BJe) in a model of LPS-stimulated THP-1 cell line, focusing on SIRT1-mediated NF-κB inhibition. We demonstrated that BJe inhibited both gene expression and secretion of LPS-induced pro-inflammatory cytokines (IL-6, IL-1β, TNF-α) by a mechanism involving the inhibition of NF-κB activation. In addition, we showed that BJe treatment reversed the LPS-enhanced acetylation of p65 in THP-1 cells. Interestingly, increasing concentrations of Sirtinol were able to suppress the inhibitory effect of BJe via p65 acetylation, underscoring that NF-κB–mediated inflammatory cytokine production may be directly linked to SIRT1 activity. These results suggest that BJe may be useful for the development of alternative pharmacological strategies aimed at reducing the inflammatory process.

## Introduction

Stimulation by pathogen-specific ligands, including bacterial lipopolysaccharide (LPS), leads cells of the innate immune system, such as monocytes and macrophages to produce several pro- and anti-inflammatory cytokines [1]. Accumulating evidences suggest that chronic inflammation represents the main contributing factor to several chronic degenerative pathologies, including cardiovascular diseases, neurological disorders and cancer [2]–[4].

Nuclear factor-kappa B (NF-κB) has been reported to play a pivotal role in inflammatory response through the induction of inflammation-related cytokines (i.e. IL-6, IL-1β, TNF-α) and enzymes such as cyclooxygenase-2 (COX-2) and inducible nitric oxide synthase (iNOS) [5], [6]. The best-studied and most prevalent form of NF-κB exists as a heterodimer composed of p50 and RelA/p65 subunits. In unstimulated cells, NF-κB is sequestered in the cytoplasm through its assembly with its inhibitory proteins, which are members of the IκB family [7], [8]. In response to various stimuli, such as cytokines, DNA-damaging agents, bacterial wall or viral proteins, IκB dissociates, via phosphorylation by the IκB kinase (IKK), and the activated transcription factor can translocate into the nucleus where is able to induce a large number of target genes involved in cell growth, apoptosis, cell adhesion and inflammation [9], [10].

According to several findings, the reversible acetylation of RelA/p65 subunit can modulate NF-κB signaling depending on the acetylation status of specific lysine residues [11]. In particular, acetylation of lysine 310 is required for full transcriptional activity of RelA and for activation of NF-κB complex [12]. Interestingly, SIRT1, a nuclear NAD^+^-dependent histone deacetylase (HDAC), may play a role in regulating inflammation by directly deacetylating the RelA/p65 protein at lysine 310. Recent studies show how the catalytic activity of SIRT1 can be modulated both positively and negatively through the binding by some agonists. Resveratrol, a polyphenolic compound found in red wine, has been identified as a potent pharmacological activator of SIRT1 [13]. On the other hand, Sirtinol was the first synthetic inhibitor extensively used in literature [14], [15].

Many anti-inflammatory drugs (SAID and NSAID) currently used in therapy target NF-κB and COX-2, but because of their frequent and serious associated side effects, result in high rates of morbidity [16], [17]. Therefore, during the last years there has been a growing interest towards the anti-inflammatory properties of some natural drugs and their bioactive compounds, like polyphenols (PP).

PP are organic molecules that constitute a numerous and heterogeneous family of secondary metabolites of plant cells, where they exert a protective action against ultraviolet radiation and oxidative stress [18]. PP are present in many edible plants and thus represent an integral part of human supply [19]. Several studies have shown that high regular intake of some phenolic compounds in the diet plays a preventive action against several human diseases, such as cardiovascular pathologies, atherosclerosis, osteoporosis, allergies, diabetes, neurodegenerative diseases and cancer [20], [21]. Although the mechanisms by which these natural compounds exert their benefits are not fully understood, anti-inflammatory effects of PP have been attributed primarily to their antioxidant activity, because they were known to scavenge and prevent the formation of reactive oxygen and nitrogen species (ROS and RNS, respectively) [22], [23], which represent important hallmarks of inflammation. Furthermore, during recent years, numerous studies have suggested that PP could influence cellular function by direct interaction with several receptors, modulation of intracellular signaling and transcription of gene involved in different pro-inflammatory pathways [24], [25].

*Citrus bergamia* Risso et Poiteau (Bergamot) is a typical fruit of the Southern Italy, belonging to the family of Rutaceae. Bergamot is cultivated almost exclusively on the Ionian coast of the province of Reggio Calabria (Italy), where there are climatic and environmental conditions particularly suitable for its cultivation. Bergamot fruit is mainly used to extract the essential oil obtained from the peel, much employed in the fragrance industry and which have been experimentally studied to evaluate its potential neuroprotective activity [26]. On the contrary, bergamot juice (BJ), obtained from the endocarp of the fruit, is considered just a secondary and discarded product. Studies performed by Miceli et al. [27] have shown that a chronic administration of BJ is effective to prevent the diet-induced hyperlipidemia in rat, suggesting a relationship between the beneficial effect and its antioxidant properties. Moreover, a clinical research showed that the bergamot-derived polyphenolic fraction, given orally in patients suffering from metabolic syndrome, produces significant reduction of serum cholesterol, triglycerides and glycaemia [28], strengthen the finding obtained in animal model. More recently, we demonstrated that BJ is also able to inhibit important molecular pathways related to cancer-associated aggressive phenotype, thus reducing growth, adhesion and migration in different *in vitro* [29] and *in vivo* models [30]. Finally, studies performed on colorectal cancer cell line verified that the antiproliferative effect of BJ is due to its flavonoid fraction which is able to act by multiple mechanisms depending on the concentration [31].

However, to date, the anti-inflammatory potential of BJ has never been evaluated. Therefore, the present study was designed to assess the modulating effects of flavonoid fraction of BJ (BJe) on the expression of inflammation-related cytokines. Considering the involvement of NF-κB pathway on the production of these pro-inflammatory mediators, we focused on the possibility that BJe may regulate NF-κB activation and examined underlying mechanisms associated with NF-κB/SIRT1 crosstalk in LPS-stimulated THP-1 monocytes, a human leukemia monocytic cell line, that have been widely used as a model to study the inflammatory cell response.

## Materials and Methods

### Materials

The human leukemia monocytic cell line, THP-1, was purchased from American Type Culture Collections (ATCC) (Rockville, MD, USA). RPMI-1640, L-glutamine, HEPES, sodium pyruvate, glucose, 2-mercaptoethanol, penicillin/streptomycin mixture, 3-(4,5-methylthiazol-2-yl)-2,5-diphenyl-tetrazolium bromide (MTT), dimethylsulfoxide (DMSO), phosphate buffered saline solution (PBS), Sirtinol and other chemicals of analytical grade were from Sigma (Milan, Italy). Lipopolysaccharide (LPS) was from InvivoGen (San Diego, CA, USA). Fetal bovine serum (FBS), as well as TRIzol for RNA extraction were from Invitrogen Life Technologies (Milan, Italy). High-capacity cDNA archive kit, TaqMan Gene Expression Mastermix, TaqMan Gene Expression assays (Assays-on-Demand) for human 18S mRNA (ID: Hs99999901_s1), TNF-α (ID: Hs00174128_m1), IL-6 (ID: Hs00985639_m1), IL-1β, (ID: Hs01555410_m1) were from Applied Biosystems (Applera Corp., Milan, Italy). Assay location (midposition of fluorogenic probe), reference sequences and other relevant information are published online by Applied Biosystems (Foster City, CA).

Instant ELISA kits for the quantitative detection of human IL-1β, IL-6 and TNF-α were from eBioscience (Vienna, Austria). Nuclear Extraction Kit and Electrophoretic Mobility Shift Assay (EMSA) for identifying NF-κB were supplied by Panomics (Santa Clara, CA, USA). Protein G PLUS-Agarose Immunoprecipitation reagent were from Santa Cruz Biotechnology (Dallas, TX, USA) and the antibodies rabbit anti-p65, sheep anti-acetylated Lysine and rabbit anti-sheep IgG (HRP) were from Abcam (Cambridge, UK). ECL Plus detection system were from GE Healthcare Bio-Sciences (Pittsburgh, PA, USA).

### Bergamot Juice extract and its chemical analysis

The flavonoid fraction of bergamot juice (BJe) has been provided by the company “Agrumaria Corleone” (Palermo, Italy). The fruits of *Citrus bergamia* were coming from crops located in the province of Reggio Calabria (Italy). The extract was centrifuged at 6000 rpm/min for 15 minutes to remove any impurities and successively transformed into a dry powder by the method of spray drying. Small aliquots of BJe were stored at −20°C. Finally, the drug was defrosted, diluted in culture media, pH adjusted to 7.4 and filtered just prior to use.

Chemical composition of BJe was investigated as previously described [32]. Briefly, BJe was solubilized in methanol to a concentration of 1 mg/mL, ultrasonicated and filtered by a 0.2 µm nylon membrane (Millipore, Milan, Italy). Qualitative and quantitative determination of flavonoids in BJe was performed using a UHPLC coupled online to an LCMS–IT-TOF mass spectrometer (Shimadzu, Kyoto, Japan). Flavonoids were identified on the basis of diode array spectra, MS molecular ions and MS/MS fragmentation patterns. Data obtained were compared with those available in scientific literature. Molecular formula was calculated by the Formula Predictor software (Shimadzu).

### Cell culture and treatment

THP-1 cells were maintained in RPMI 1640 supplemented with L-glutamine (2 mM), HEPES (10 mM), sodium pyruvate (1 mM), glucose (2.5 g/l), 2-mercaptoethanol (0,05 mM), 10% heat-inactivated fetal bovine serum (FBS), 1% penicillin/streptomycin, at 37°C in a 5% CO_2/_95% air humidified atmosphere. Medium was renewed every 2 days and split performed when cells reached maximum density (1×10^6^ cells/ml). In our experimental conditions, THP-1 cells were seeded at a density of 5×10^5^ cells/ml into culture plates in RPMI complete medium plus 10% FBS and incubated at 37°C with lipopolysaccharide (LPS; 500 ng/ml) for 3 hs, in the presence or absence of BJe (0.05–0.1–0.5 mg/ml) and/or Sirtinol (1–5–10 µM), which were added to the culture medium 30 min prior to LPS treatment. In all experiments, equal volumes of PBS or DMSO were added to the medium of control cultures (controls were performed using non-stimulated cell). Either concentrations of LPS, BJe and Sirtinol were chosen according to our preliminary optimization studies. After incubation, cells were harvested by centrifugation to assess cellular viability, gene expression, activation of transcription factor NF-κB and acetylation status of p65. Media were collected in order to evaluate cytochine release.

### Cell viability assay

To assess either LPS and BJe adverse effects on cell viability, we evaluated the mitochondrial activity of living cells by a MTT quantitative colorimetric assay. After treatment, THP-1 cells were harvested by centrifugation and, after counting, they were incubated in 96-well culture plates at a density of 5×10^4^ cells/well with fresh red-phenol free medium containing MTT (0.5 mg/mL) at 37°C for 4 hs. Then, insoluble formazan crystals were dissolved in 100 µL of a 0.04 N HCl/isopropanol solution for 1 h. The optical density in each well was evaluated by spectrophotometrical measurement. Absorbance was determined at 570 nm using a microplate reader (Tecan Italia, Cologno Monzese, Italy). All experiments were performed in eightplicate and repeated three times.

### Real-Time PCR

After RNA isolation with TRIzol reagent, RNA (3 µg) was reverse transcribed with High- Capacity cDNA Archive kit according to the manufacturer's instructions. Then, mRNA levels of IL-6, IL-1β, TNF-α were analyzed by real-time PCR using TaqMan gene expression assays according to the manufacturer's instructions. 18S mRNA was used as endogenous controls. Quantitative PCR reactions were set up in triplicate in a 96-well plate and were carried out in 10 µl reactions containing 1× TaqMan Gene Expression Mastermix, 1× TaqMan-specific assay and 20 ng RNA converted into cDNA. qPCR was performed in a 7900HT Fast Real-Time PCR System with the following profile: one cycle at 50°C for 2 min, then 95°C for 10 min, followed by 50 cycles at 95°C for 15 s and 60°C for 1 min. Data were collected and analyzed using SDS 2.3 and RQ manager 1.2 software (Applied Biosystems, Foster City, CA) using the 2^(−ΔΔCt)^ relative quantification method. Values are presented as fold change relative to unstimulated cells.

### Evaluation of cytokine secretion by ELISA

In order to detect human IL-6, IL-1β and TNF-α, an enzyme-linked immunosorbent assay was performed in cell-free culture supernatants of THP-1 monocytes, using Instant ELISA Kits. Before detection, supernatants recovered from treated and untreated cells were concentrated 10-fold by freeze-drying. All freeze-dried samples were reconstituted by the addition of distilled water. Briefly, according to the manufacturer's guidelines, 50 µl of standards or samples (supernatants recovered from treated and untreated cells) were incubated in 96-well plates at room temperature for 3 hs with shaking. After washing 5 times with 400 µl of wash buffer, 100 µl of the provided substrate solution were added to each well and the plates were incubated in the dark for 10 min. The enzyme reaction was then stopped by pipetting 100 µl of stop solution into each well and the absorbance was determined at 450 nm using a microplate reader (Tecan, Italy). All experiments were performed in triplicate.

### Electrophoretic mobility shift assay

At the end of the treatment, THP-1 cells were harvested by centrifugation. After washing twice with cold PBS, the isolation of nuclear cell proteins was performed using a commercial nuclear extraction kit following the manufacturer's guidelines. Protein concentrations were determined using a Bradford method. The presence of NF-κB DNA binding activity in cellular nuclear extracts of LPS-treated and control cells was evaluated by subsequent electrophoretic mobility shift assay, using Affymetrix EMSA Kits according to the manufacturer's instructions. Briefly, nuclear extracts were incubated with the biotin-labeled NF-κB probe and then the protein/DNA complexes were separated on a non-denaturing 6% polyacrylamide gel. After transferring onto nylon membranes bound complexes were detected via streptavidin-HRP and a chemiluminescent substrate and visualized on Kodak film. The bands were scanned and quantified by densitometric analysis with ImageJ 1.47, an open source software freely downloadable from the US National Institute of Health website (http://imagej.nih.gov/ij/).

### Immunoprecipitation and immunoblotting analyses

For each sample, 50 µg of nuclear extract were incubated with rabbit anti-p65 for 1 h at 4°C on a rotator. Negative control was set by incubating nuclear proteins under similar conditions but without the immunoprecipitating antibody. Afterwards, 35 µl of resuspended Protein G PLUS-Agarose beads were added to each tube and the samples were incubated at 4°C overnight on a rocker platform. The agarose beads were extensively washed the next day and pellets were resuspended in 40 µl of 1x Laemmli buffer, boiled for 5 min and resolved by SDS-PAGE. Proteins were then transferred onto nitrocellulose membrane and non-specific binding sites were pre-blocked by incubation with 5% non-fat dry milk in Tris-buffered saline containing 0.15% Tween 20 for 1 h at room temperature. The blot was probed overnight at 4°C with primary antibody anti-acetylated Lysine (from sheep, diluted 1∶1000), followed by incubation for 2 hs with horseradish peroxidase-conjugated anti-sheep secondary antibody (diluted 1∶3000). Final detection was performed by using ECL chemiluminescence system; then, bands were scanned and quantified by densitometric analysis with ImageJ software.

### Statistical analysis

Data obtained from three separate experiments were expressed as mean ± SEM, and analyzed by one-way analysis of variance (ANOVA) and the Student-Newman Keuls test using GraphPad Prism software (San Diego, CA). p values lower than 0.05 were considered significant.

## Results

### The flavonoid profile of the BJe

**Figure 1:**
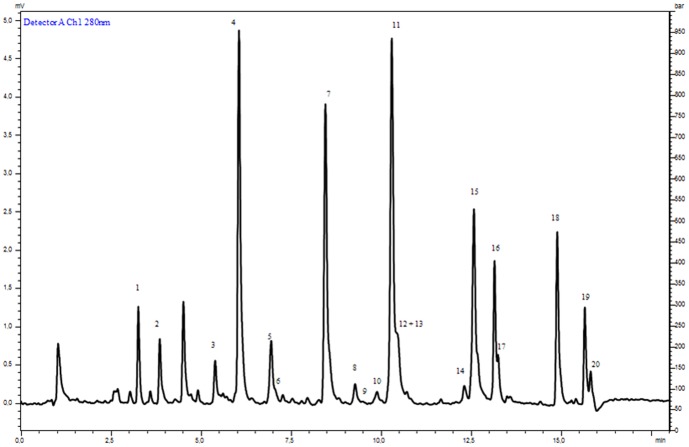
UHPLC chromatogram of BJe. A representative chromatogram of the BJe flavonoid components is shown. The sample was run for five times. For peak identification see Table 1.

A chromatogram of the BJe composition is shown in figure 1. Peaks 1–20 correspond to identified flavonoid present in the extract and their amounts are showed in table 1. The main flavonoids identified (mg/g) were Neohesperidin (105.27), Naringin (101.88), Melitidin (75.89), Neoeriocitrin (56.61) and Hesperetin (55.65). Quantitative values were obtained as the average of quintuplicate analyses and were in accordance with tipical BJ flavonoid profile reported in the literature [27], [32].

**Table 1:** Flavonoid concentration (mg/g) in BJe.

Peak	Compound	Synonyms	Quantity mg/g
**1**	Apigenin 6,8 di C-glucoside	Vicenin-2	11.98
**2**	Diosmetin 6,8 di C-glucoside	Lucenin-2 4′-methyl ether	11.34
**3**	Eriodictyol 7-O-rutinoside	Eriocitrin	8.08
**4**	Eriodictyol 7-O-neohesperidoside	Neoeriocitrin	56.61
**5**	5,7-dihydroxy-4′ methoxyflavone 7-O-rutinoside	Poncirin	17.01
**6**	Diosmetin 8-C-glucoside	Orientin 4′ methylether	15.06
**7**	Naringenin 7-O-neohesperidoside	Naringin	101.88
**8**	Apigenin 7-O-neohesperidoside	Rhoifolin	18.00
**9**	Hesperetin-7-O-rutinoside	Hesperidin	8.37
**10**	Quercetin-3-β-glucopyranoside	Isoquercitrin	2.05
**11**	Hesperetin-7-O-neohesperidoside	Neohesperidin	105.27
**12**	Diosmetin 7-O-neohesperidoside	Neodiosmin	13.40
**13**	Apigenin 7-O-neohesperidoside-4′-glucoside	Rhoifolin 4′-glucoside	1.18
**14**	Naringenin-7-O-rutinoside	Narirutin	6.02
**15**	Naringenin 7-[2”-α-rhamnosyl-6”-[3””-hydroxy-3””-methylglutaryl]-β-glucoside]	Melitidin	75.89
**16**	Hesperetin 7-[2”-α-rhamnosyl-6”-[3””-hydroxy-3””-methylglutaryl]-β-glucoside]	Brutieridin	21.99
**17**	unknown		10.55
**18**	5,7-dihydroxy-2-(4-hydroxyphenyl)chroman-4-one	Naringenin	43.22
**19**	(S)-2,3-dihydro-5,7-dihydroxy-2-(3-hydroxy-4-methoxyphenyl)-4H-1-benzopyran-4-one	Hesperetin	55.65
**20**	5,7-dihydroxy-2-(3-hydroxy-4-methoxyphenyl)chromen-4-one	Diosmetin	14.01

### Expression of LPS-induced proinflammatory cytokines in presence of BJe

**Figure 2:**
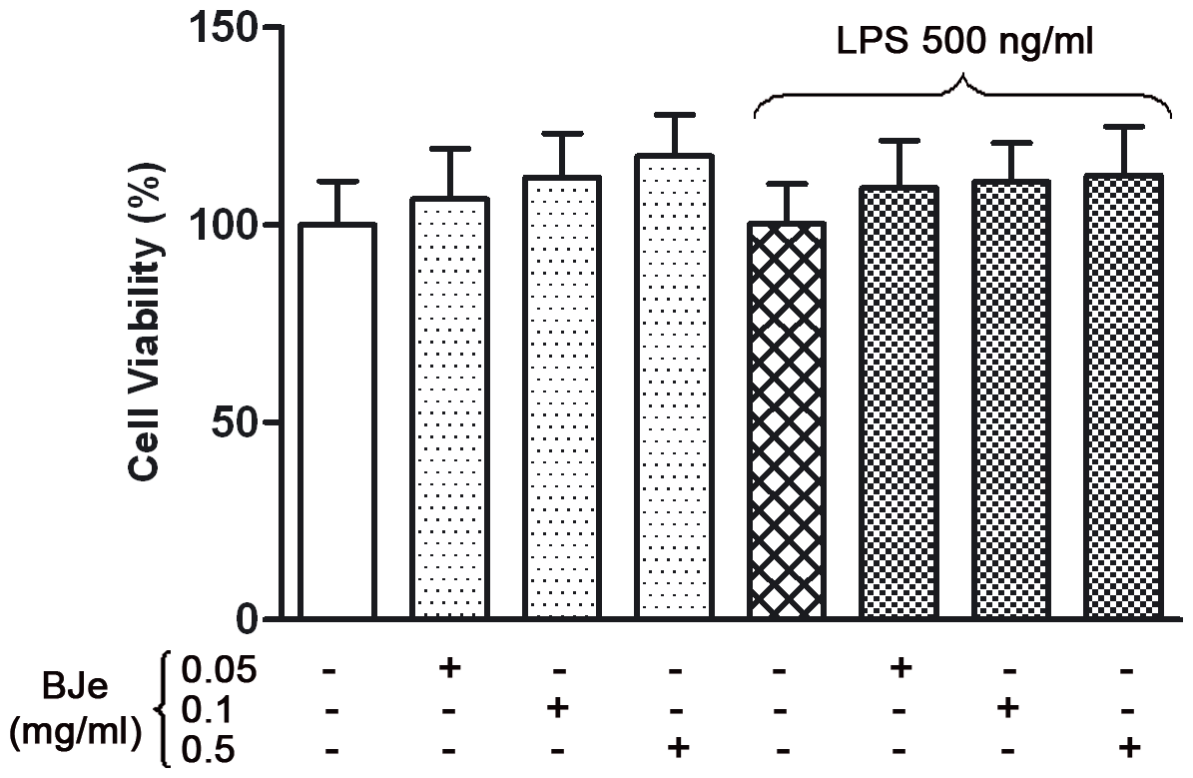
Effect of BJe on THP-1 cell viability in presence or absence of LPS. Different concentrations of BJe (0.01, 0.05, 0.1 and 0.5 mg/ml) were added to the culture medium 30 min before LPS treatment (500 ng/ml for 3 hs) and then cell viability was assessed by the MTT test. Results are expressed as percentages of untreated cultures. Data are means ± SEM from three independent experiments performed in eightplicate.

In order to assess BJe toxicity in cell cultures, preliminary experiments were carried out using MTT test. Exposure of THP-1 monocytes to different BJe concentrations (in a range 0.05–0.5 mg/ml) for 3.5 hs didn't show any significant reduction of cell viability when BJe was added in presence or absence of LPS, as shown by MTT data (Fig. 2).

**Figure 3:**
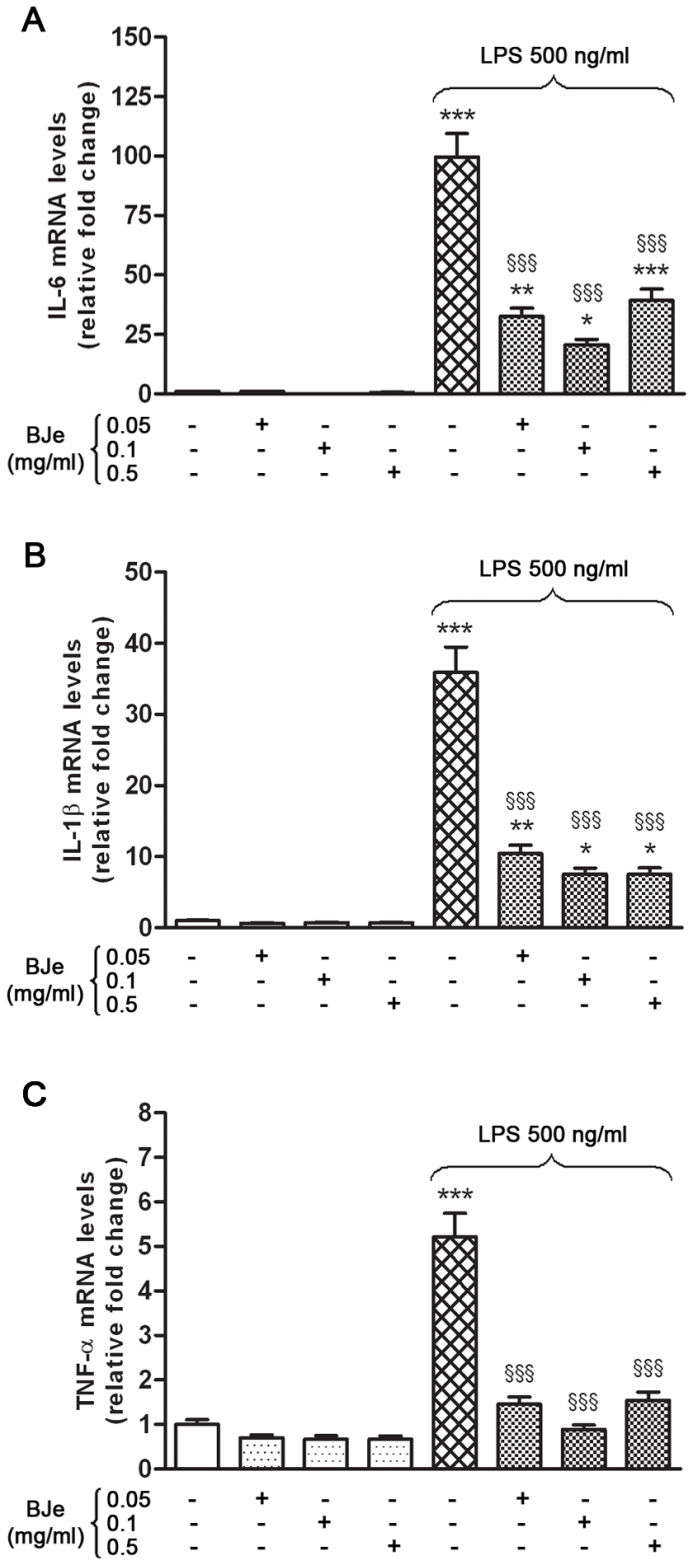
Effects of BJe on cytokine gene expression in THP-1 cells stimulated with LPS. THP-1 cells were treated with different concentrations of BJe (0.05–0.5 mg/ml for 30 min) before exposure to 500 ng/ml of LPS for 3 hs. Results from real-time PCR of IL-6 (A), IL-1β (B) and TNF-α (C) are expressed as a relative fold change compared to untreated cells, after normalization against 18S as endogenous control. Columns and bars represent means ± SEM from triplicate experiments. * p<0.05, ** p<0.01, *** p<0.001, significant values in comparison with control cells; ^§§§^ p<0.001, significant values in comparison with LPS treated cells (ANOVA followed by Student-Newman Keuls multiple comparisons test).

Based on preliminary experiments we used LPS at the concentration of 500 ng/ml that was able to trigger the release of pro-inflammatory factors in THP-1 cells within three hours. Under these conditions, the Real-time PCR analysis showed that LPS stimulation of THP-1 monocytes resulted in a dramatic increase of mRNA of pro-inflammatory cytokines (IL-6, IL-1β, TNF-α), relative to endogenous 18S mRNA levels. In particular, IL-6 mRNA increase was higher than those observed for IL-1β and TNF-α. The IL-6 mRNA levels in LPS-treated cells was almost one hundred-fold higher than those found in untreated cells (Fig. 3A). These effects were reduced in presence of BJe. Specifically, LPS-induced IL-6 up-regulation was strongly reduced in presence of BJe 0.05 mg/ml (by ∼70%), with a maximum reduction of ∼80% with 0.1 mg/ml of BJe (p<0.001). Even though to a lesser extent, also BJe 0.5 mg/ml was able to reduce IL-6 mRNA levels (∼60%; p<0.001). In parallel, the LPS-induced increase in IL-1β gene expression (36-fold higher than controls) was significantly reduced by all BJe concentrations used (from 70 to 80%; p<0.001; Fig. 3B). In a similar way, stimulation of THP-1 monocytes with LPS caused a 5-fold increase of TNF-α mRNA levels in comparison with untreated cells, while a significant down-regulation of LPS-stimulated TNF-α gene expression was observed in presence of BJe treatment (in the range 0.05–0.5 mg/ml), resulting in mRNA levels similar to those found in control cells (Fig. 3C).

### The release of cytokines caused by LPS was reduced by BJe treatment

**Figure 4:**
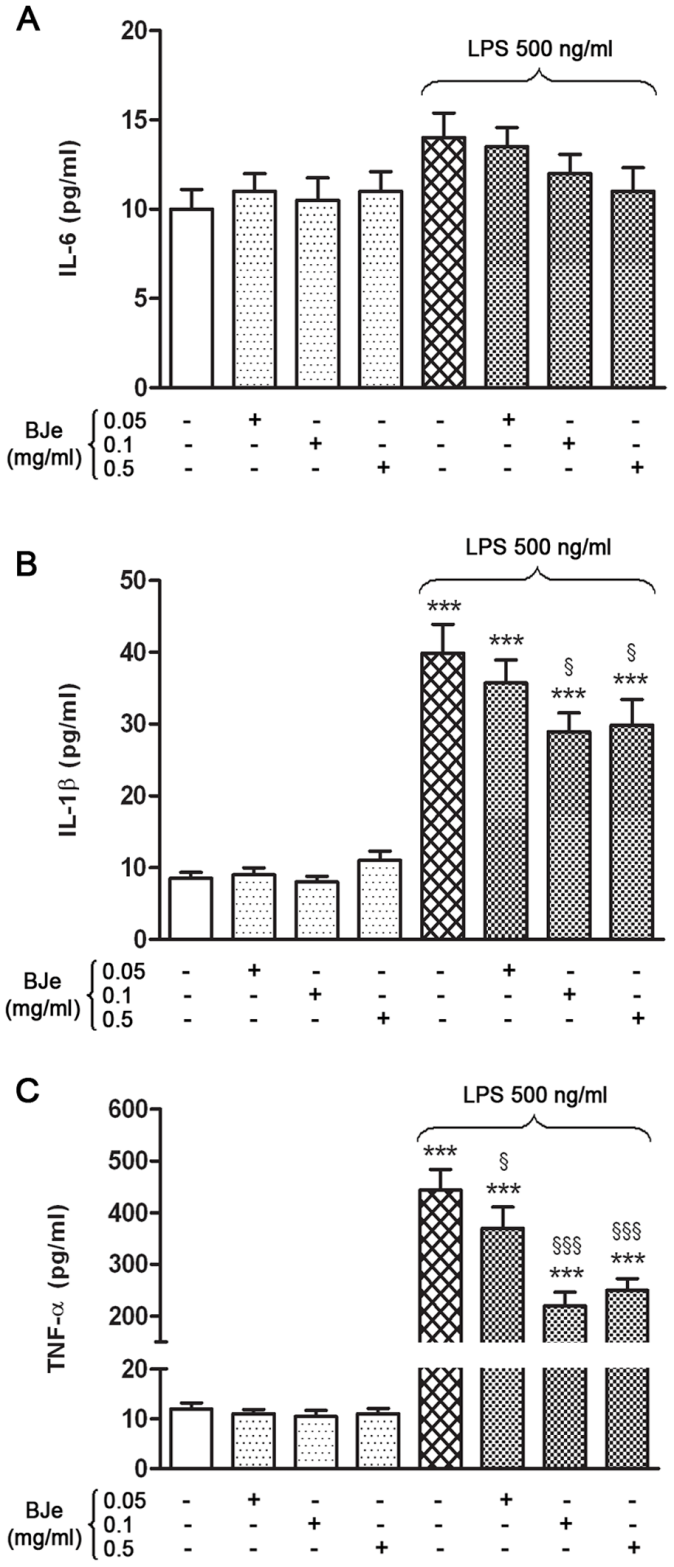
BJe prevents the LPS-stimulated release of IL-1β and TNF-α in THP-1 monocytes. The cells were treated with increasing concentrations of BJe (0.05–0.5 mg/ml for 30 min) prior to add LPS (500 ng/ml; 3 hs). Then, secretion of IL-6 (A), IL-1β (B) and TNF-α (C) in the media was evaluated by ELISA assay. Data are the mean ± SEM of three independent experiments performed in triplicate. *** p<0.001, significant differences *vs* untreated cultures; ^§^ p<0.05 and ^§§§^ p<0.001, significant differences *vs* LPS treated cells (ANOVA followed by Student-Newman Keuls multiple comparisons test).

In order to confirm the LPS-induced up-regulation of pro-inflammatory genes, we assessed the release of the analyzed cytokines by detection in the supernatants collected at the end of the treatment. As shown in figure 4, in comparison to untreated cells, 3 hs of LPS stimulation was able to trigger the secretion of significant amount of IL-1β and especially of TNF-α (4,7 and 37 fold increases, respectively) in THP-1 monocytes, but not of IL-6. These LPS-induced releases were reduced by BJe treatment. In particular, both BJe 0.1 and 0.5 mg/ml decreased significantly the amount of IL-1β secreted by 27 and 25%, respectively (p<0.05 *vs* LPS-treated cultures; Fig 4B), while all BJe concentrations used affected the release of TNF-α between 17 and 50% (p<0.05 and p<0.001 *vs* LPS-treated cells; Fig 4C). Interestingly, the most effective concentration of BJe able to diminish the protein levels of both IL-1β and TNF-α was 0.1 mg/ml.

### Inhibition of LPS-induced NF-κB activation by BJe

**Figure 5:**
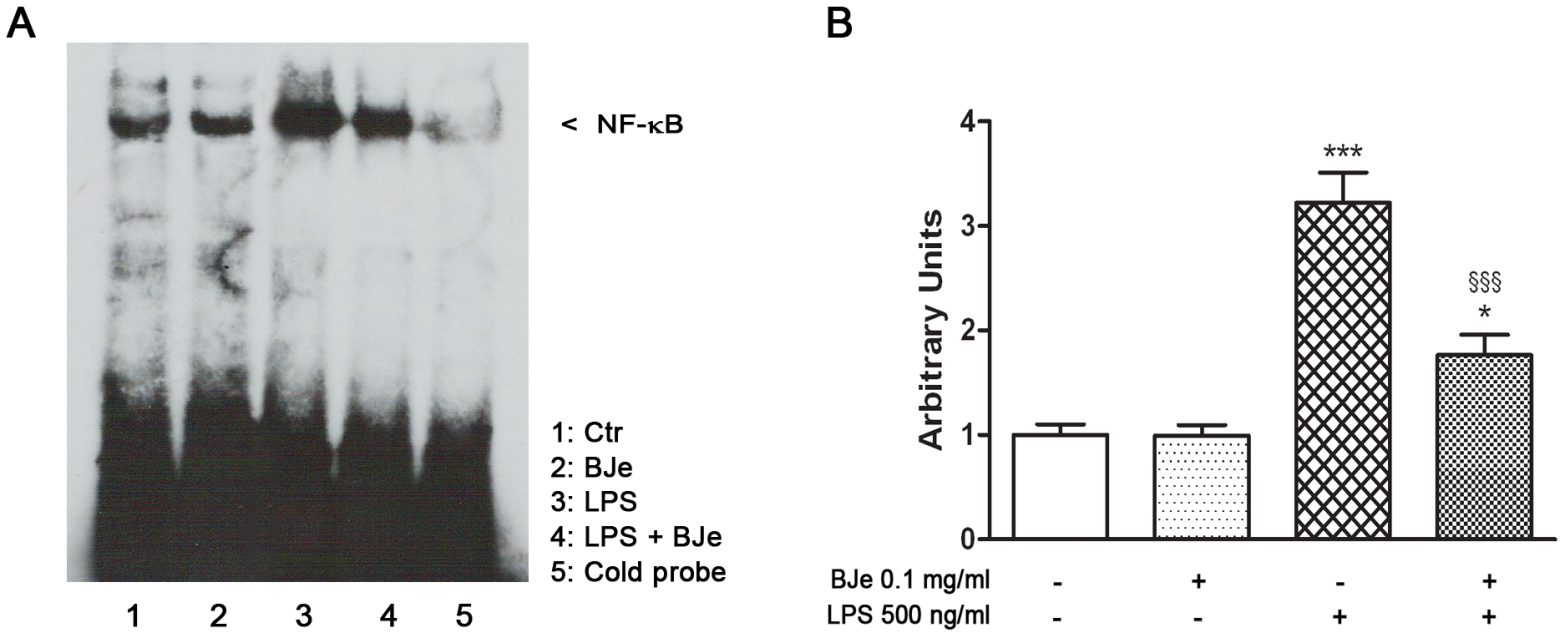
Inibitory effect of BJe on LPS-induced NF-κB activation in THP-1 cells. (A) The cells were exposed to 0.1 mg/ml BJe 30 min before LPS treatment (500 ng/ml for 3 hs), and then NF-κB activation was determined by the electrophoretic mobility shift assay (EMSA). A competition assay was performed using both biotin-labeled and unlabeled specific probe (cold probe, CP). (B) Densitometric analysis of three independent blots (mean ± SEM) is reported. * and *** p<0.05 and p<0.001 *vs* untreated cultures, respectively; ^§§§^, p<0.001 *vs* LPS-treatment (ANOVA followed by Student-Newman Keuls multiple comparisons test).

Given the reported pivotal role of NF-κB in inflammatory response induced by various stimuli, we also investigated its role in THP-1 cell response to LPS-induced injury, in presence of the most effective concentration of BJe (0.1 mg/ml). EMSA analysis of nuclear fractions showed that nuclear translocation and specific DNA binding activity of NF-κB increased in THP-1 cell cultures, after 3 hs of incubation with LPS (Fig. 5). Interenstingly, BJe 0.1 mg/ml was able to inhibit the LPS-induced NF-κB activation.

### Involvement of SIRT1 in the inhibition of NF-κB signaling exerted by BJe, via the p65 acetylation

**Figure 6:**
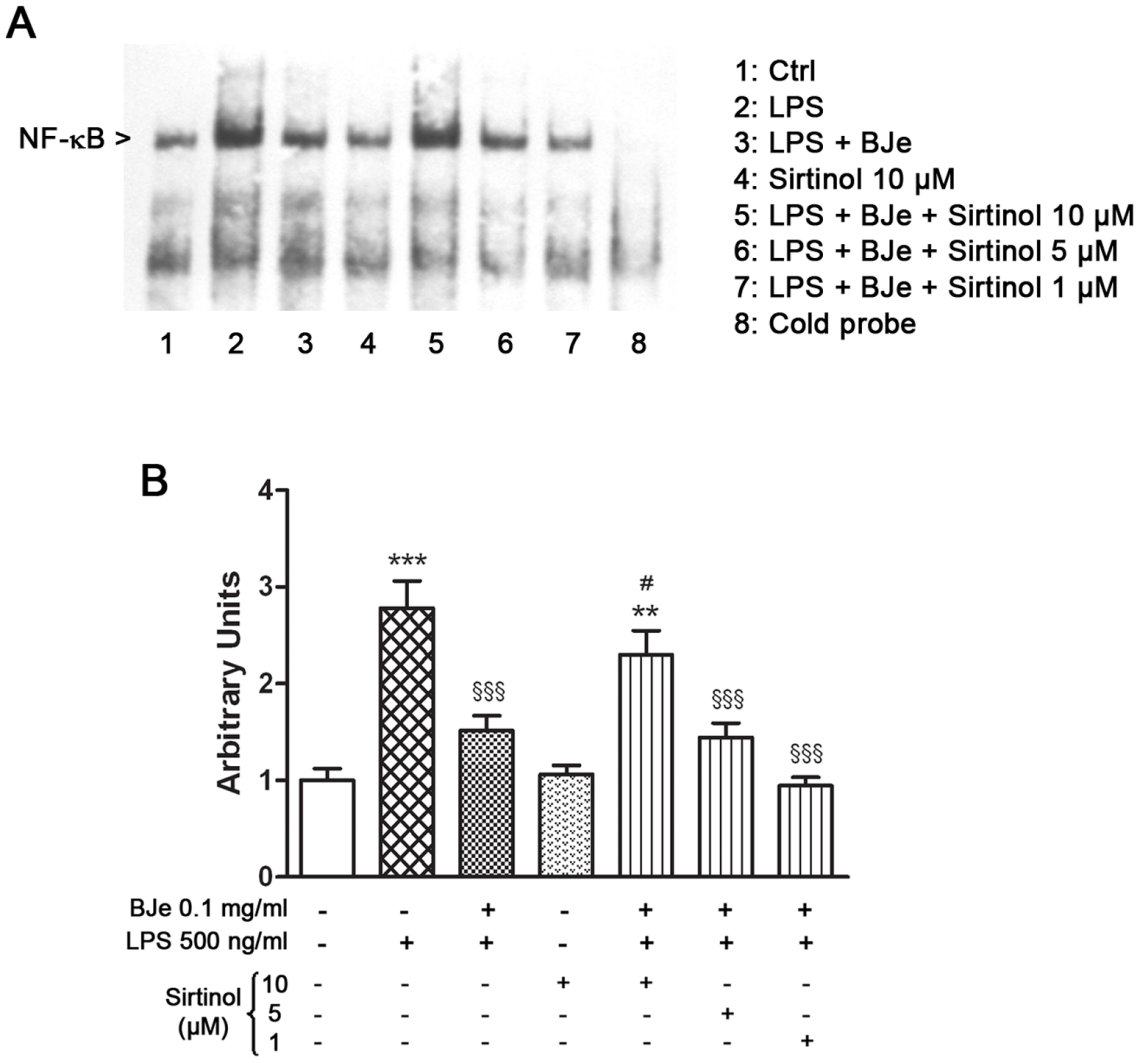
Sirtinol reverts the inhibitory effect of BJe on LPS-induced activation of NF-κB. (A) Exposure of THP-1cells to 0.1 mg/ml BJe reduced LPS-induced NF-κB activation; this effect was reverted by 10 µM Sirtinol, a SIRT1 inhibitor. EMSA analysis was performed using both biotin-labeled and unlabeled specific probe (cold probe). (B) Densitometric analysis of three independent blots (mean ± SEM) is presented. ** p<0.01 and *** p<0.001 *vs* controls; ^§§§^, p<0.001 *vs* LPS treated cells. ^#^ p<0.05 *vs* BJe plus LPS treated cells (ANOVA followed by Student-Newman Keuls multiple comparisons test).

Since it is known that SIRT1 may suppress inflammation by deacetylation of NF-κB subunit, in a subset of experiments we also investigated its regulatory effects on NF-κB activation. As shown in fig. 6, the inhibitory effect of 0.1 mg/ml BJe on the LPS-induced activation of NF-κB was counteracted at highest dose of Sirtinol, the well known inhibitor of SIRT1. In particular, 10 µM of Sirtinol reverted the NF-κB inhibition due to BJe treatment, suggesting a role of SIRT1 in the modulatory effect of BJe on NF-κB signaling.

**Figure 7:**
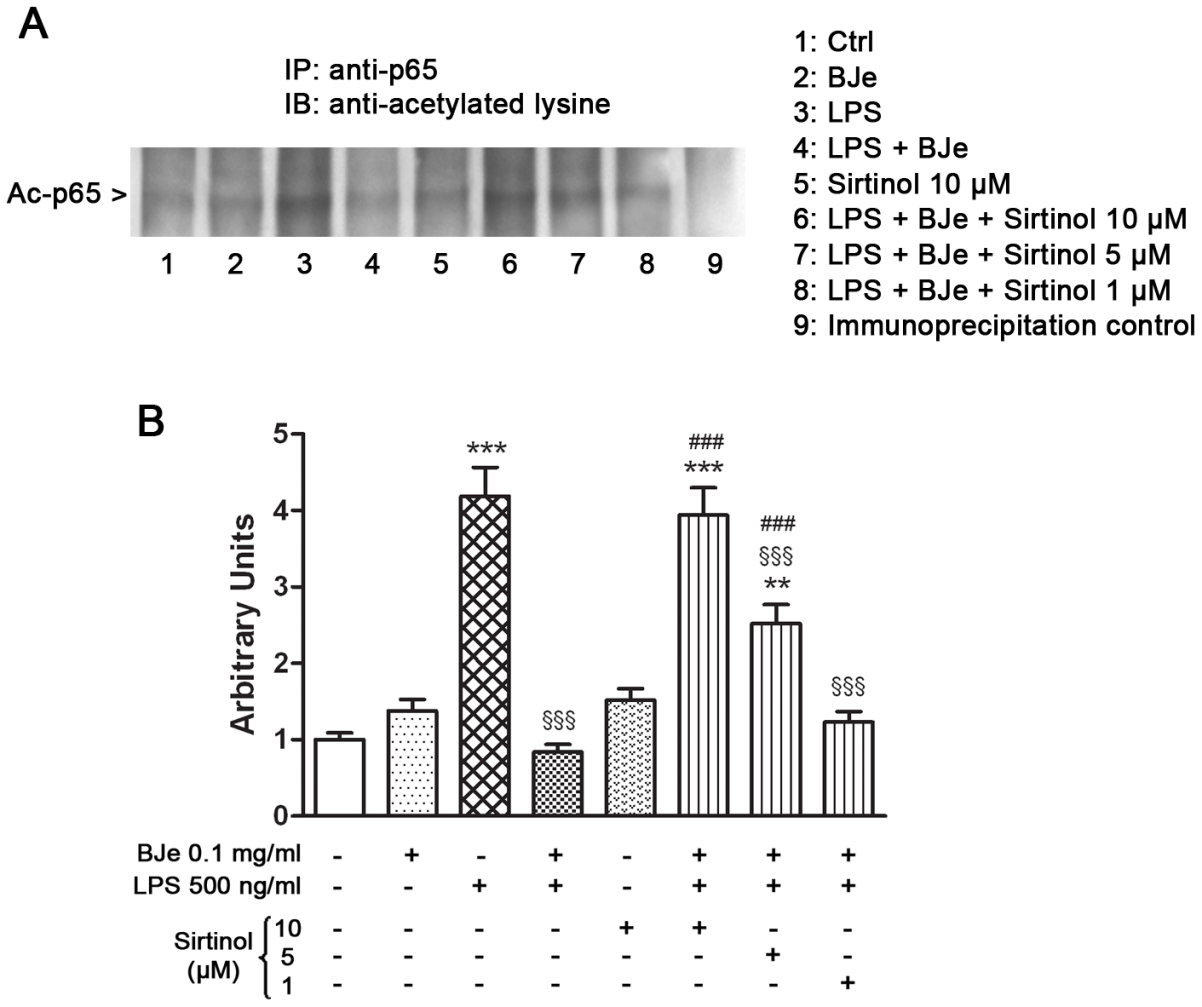
BJe treatment reverts the LPS-enhanced acetylation of p65 in THP-1 cells. (A) After LPS treatment in presence or absence of BJe and/or Sirtinol, THP-1 cells were lysed and proteins were immunoprecipitated using an anti-p65 antibody. Immunoprecipitated proteins were separated by SDS-PAGE and immunoblotted with antibody against acetyl-lysine residues. Immunoprecipitation negative control was set by incubating cell lysates under similar conditions, but without the immunoprecipitating antibody. (B) Densitometric analysis of three independent blots (mean ± SEM) is presented. ** p<0.01 and *** p<0.001 *vs* control cultures; ^§§§^ p<0.001 *vs* LPS treated cells; ^###^ p<0.001 *vs* LPS plus BJe treated cells (ANOVA followed by Student-Newman Keuls multiple comparisons test).

In order to clarify the involvement of SIRT1's deacetylating activity and the molecular mechanism underlying the BJe-mediated inhibition of NF-κB, an immunoprecipitation assay was carried out to evaluate the acetylation status of RelA/p65 subunit. As shown in Fig.7, BJe 0.1 mg/ml treatment reverted the LPS-enhanced acetylation of p65 in THP-1 monocytes. In addition, increasing concentrations of Sirtinol were able to suppress the inhibitory effect of BJe on the activation of NF-κB, via p65 acetylation, underscoring that NF-κB–mediated inflammatory cytokine production may be directly linked to SIRT1 activity.

## Discussion

Several results show that THP-1 monocyte/macrophages are a sensitive *in vitro* model to analyze potential anti-inflammatory activity of different substances, so that it may be a reasonably accurate model to study LPS-dependent inflammatory response [33]–[35]. Thus, THP-1 cells is considered a suitable and reliable model for screening a variety of compounds prior to a more detailed analysis with human derived cells. According to other results [36], [37], the gene expression of pro-inflammatory cytokines such as IL-1β, TNF-α and IL-6 increased within the first hours of the LPS challenge, whereas changes in the secretion pattern of related cytokines did not show similar extent. However, differences between amount of cytokines secreted from THP-1 monocytes and mRNA levels of corresponding genes can be also explained by differences in RNA stability, post-translational modification factors and proteolytic processing events that make the production of individual cytokines different [38]. Under our conditions, the secreted amount of both TNF-α and IL-1β, but not that of IL-6, were higher than controls, suggesting a general relation between mRNA and protein levels in LPS-stimulated monocyte/macrophages. In this study for the first time we demonstrated that the flavonoid fraction of Bergamot juice is able to reduce significantly both transcription profile and protein levels of pro-inflammatory cytokines. Furthermore, we reported a concentration-dependent effect of BJe in a range useful to exclude toxic effects in THP-1 cultures.

As previously reported [6], the induction of most genes involved in the inflammatory response was abolished or attenuated under the inactivation of IκB kinase/nuclear factor-κB (IKK/NF-κB) pathway. NF-κB plays a crucial role in coordinating cellular response to infections, stress and injury. When the inhibitory protein IκB dissociates, the most common active heterodimer, dealing with RelA/p65 and p50, is able to trigger both innate and adaptive immune responses through the induction of several pro-inflammatory genes [5]. Furthermore, RelA acetylation increases transcriptional activity of NF-κB [39], which in turn regulates genes encoding cytokines and metalloproteases. On the other hand the deacetylation of RelA promotes its effective binding to IκBα leading to IκBα-dependent nuclear export of NF-κB. Indeed, once in the nucleus, the activated transcription factor associates with several histone acetyltransferases (HATs) that catalyze the acetylation of RelA/p65 and lead to “open chromatin” configuration through the acetylation of N-terminal tails of histones.

In this way, the acetylation of specific lysine residues can affects both the DNA-binding ability and transcriptional activity of the protein [12], [17].

Therefore, NF-κB is a target for many anti-inflammatory drugs and during the last years several studies have focused on polyphenols, natural compounds that would act as activators of SIRT1, a member of the class III HDAC family that has been implicated in modulating epigenetic gene silencing and cell survival, via acetylation of several both histonic and non-histonic substrates. Indeed several recent studies confirmed that SIRT1-mediated deacetylation of p65/RelA inhibited the NF-κB signaling and the activation of SIRT1 could alleviate a multitude of NF-κB-driven inflammatory and metabolic disorders [40], [41]. This implies that SIRT1 activators could exert significant benefits in the treatment of inflammation.

Several findings have shown that polyphenols show anti-inflammatory activity in both *in vitro* and *in vivo* models, by modulation of pro-inflammatory gene expression such as COX-2, iNOS and several pivotal cytokines [42], [43]. Due to their properties, flavonoids might be reasonable candidates for the development of new anti-inflammatory drugs, although their mechanism of action remains not fully understood.

On the basis of these results, we investigated the modulatory effect of BJe on a LPS-induced inflammatory response, focusing on SIRT1-mediated NF-κB inhibition. Our results provide evidence for BJe impact on NF-κB pathway, likely via SIRT1 activation. In order to confirm our hypothesis we tested the effects of Sirtinol, a synthetic molecule that inhibits SIRT1 functionality by occupying the site which normally functions as the binding site for the adenine base of NAD+ [15]. Under our conditions, we observed that Sirtinol triggered RelA/p65 binding in the nuclear fraction of LPS-activated cells. Finally, immunoprecipitation assay suggested that Sirtinol and BJe produced opposite effects on SIRT1's deacetylating activity. Considering the Sirtinol-mediated inhibition of SIRT1 and that BJe could act as SIRT1 activators, these results underscore at molecular levels that the BJe-mediated inhibition of NF-κB may be associated to acetylation status of p65/RelA subunit.

Flavonoids are widely recognized as naturally occurring antioxidants and several protective effects against cell injury have been associated to their antioxidant properties. Here we also demonstrated that bioactive molecules present in the BJe are responsible of specific inhibition of intracellular pathway involved in NF-κB-mediated inflammatory response. Noteworthy, our results have been obtained by use of a phytocomplex rather than those of individual components. According other results [27] we believe that the complex mixtures of bioactive molecules present in BJe could be more effective than their individual constituents to induce beneficial effects through both additive and synergistic action.

Our study, for the first time, shows the *in vitro* anti-inflammatory activity of flavonoid fraction from bergamot juice, suggesting the activation of SIRT1 could be a relevant target for novel therapeutic approaches. In addition, our observations (i) demonstrate the inhibitory effects of BJe on LPS-induced increases in mRNA transcripts and protein levels of pro-inflammatory cytokines, (ii) confirm the presence of SIRT1/NF-κB cross-talk in a model of inflammation such as THP-1 monocyte/macrophages treated with LPS, (iii) give evidence for the involvement of SIRT1 in the production and secretion of cytokines, representing the first data on the interplay existing between SIRT1, NF-κB and BJe effects in LPS-stimulated THP-1 cells.
